# Slow-Channel Congenital Myasthenic Syndrome due to a CHRNA1 Variant Masquerading as Atypical Anorexia Nervosa: A Case Report

**DOI:** 10.7759/cureus.96271

**Published:** 2025-11-06

**Authors:** Timothy Ming Him Yeung, Yuan Gao, Chun Fung So, Tsz Ki Ling, Ching Wan Lam

**Affiliations:** 1 Division of Chemical Pathology, Department of Pathology, Queen Mary Hospital, Hong Kong, HKG; 2 Division of Neurology, Department of Medicine, Queen Mary Hospital, School of Clinical Medicine, The University of Hong Kong, Hong Kong, HKG; 3 Department of Pathology, The University of Hong Kong, Hong Kong, HKG

**Keywords:** anorexia nervosa (an), chrna1, congenital myasthenic syndrome (cms), low body weight, neurogenetic, neuromuscular junction disorders, slow-channel congenital myasthenic syndrome (sccms)

## Abstract

Congenital myasthenic syndrome (CMS) is a group of inherited disorders characterized by skeletal muscle weakness resulting from dysfunction of the neuromuscular junction. Here, we report a case of a 42-year-old woman with slow-channel congenital myasthenic syndrome (SCCMS) whose phenotype included low body weight, which mimicked anorexia nervosa. The patient experienced progressive weight loss from 45kg (BMI 18.3) to 34kg (BMI 13.8) during her 20s despite not engaging in dieting, purging, or excessive exercising, nor did she express a desire to lose weight. The patient had asymmetrical fatigable weakness since the age of 10. Electrophysiology studies identified repetitive compound motor action potentials on nerve stimulation. Heterozygous NM_000079.4(*CHRNA1*):c.737C>T p.(Ser246Phe), a pathogenic variant for SCCMS, was detected by Sanger sequencing. This case illustrates the use of electrophysiology studies and genetic testing in differentiating organic causes of low body weight from anorexia nervosa.

## Introduction

Congenital myasthenic syndrome (CMS) is a heterogeneous group of inherited disorders that manifests as skeletal muscle weakness as a result of dysfunction of the neuromuscular junction. Slow-channel congenital myasthenic syndrome (SCCMS) in particular is characterized by prolonged duration of end-plate potentials due to abnormal response of the acetylcholine receptor (AChR) to acetylcholine (ACh) [1]. SCCMS can be identified through characteristic electrophysiological findings and confirmed by genetic analysis. While SCCMS may mimic myasthenia gravis (MG), it can be distinguished from autoimmune MG by the presence of repetitive compound motor action potential (CMAP) on electrophysiological testing [2], pathogenic variants of relevant genes, poor response to acetylcholinesterase (AChE) inhibitor medications [3], and clinical improvement to channel blocking agents such as fluoxetine and quinidine [3,4]. Here, we report a case where a patient with SCCMS demonstrated a phenotype of low body weight, mimicking anorexia nervosa (AN). This illustrates the importance of a high index of suspicion for rare neuromuscular diseases and the role of genetic testing in patients with features of AN.

## Case presentation

A 42-year-old Chinese woman presented to Neurology for slowly progressive generalized limb weakness. The patient experienced the onset of symptoms at 10 years old, with progressive fatigable weakness over the years that is more severe during winter. The patient remained ambulatory without aid and coped well with her daily activities. The patient’s younger sister and father also experienced similar symptoms of generalized progressive weakness.

**Figure 1:**
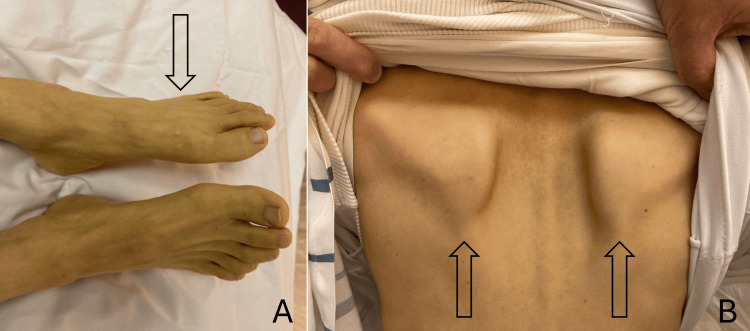
(A) Physical examination revealed asymmetrical muscle wasting more prominent on the left foot and (B) winging of the scapula.

Physical examination of the patient revealed asymmetrical weakness of the limbs that is most severe over the left distal lower limb, with left ankle dorsiflexion power 0/5 on the Medical Research Council (MRC) scale. Less severe weakness was also present over her proximal left lower limb, right lower limb, and bilateral upper limb (MRC grade 4/5). Muscle wasting was observed over the limbs, most severe over the left lower limbs (Figure 1A). There was also winging of the scapula (Figure 1B), facial weakness (House Brackmann grade II), dysarthria, and ophthalmoplegia over right lateral conjugate gaze without nystagmus. Gait examination revealed left foot drop, but was otherwise unremarkable. Sensory, reflexes, and coordination were normal on physical examination.

The patient experienced gradual progressive weight loss from 45kg (BMI 18.3) to 34kg (BMI 13.8) over a period of approximately 10 years during her 20s and early 30s. She was referred to Psychiatry for abnormal body weight at 31 years old and was diagnosed to have atypical anorexia nervosa by the International Classification of Diseases, Tenth Revision (ICD-10) criteria [5]. Her body weight remained between 33kg to 35kg from 31 years old to 42 years old. She reported eating regularly, though her weakness limited the amount of food she ate at each meal. She did not engage in dieting, purging, or excessive exercising. She has no abnormal body image issues nor a fear of gaining weight. On the contrary, she reported a desire to gain weight. She had normal and regular menses.

**Figure 2:**
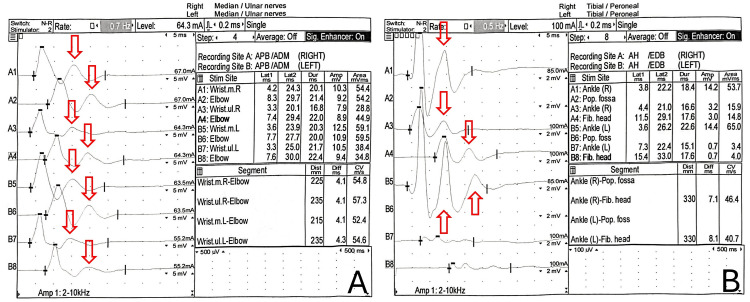
Nerve stimulation study of the (A) bilateral median and ulnar nerves, and (B) bilateral tibial and peroneal nerves. Single stimulation of the bilateral median, ulnar, tibial, and peroneal nerves elicited repetitive CMAPs, characteristic of SCCMS. CMAP: compound motor action potential; SCCMS: slow-channel congenital myasthenic syndrome

Electrophysiological studies were conducted by Neurology to investigate the patient's weakness. Nerve stimulation study on the patient, who was 42 years old at the time, demonstrated a repetitive compound motor action potential (CMAP) on single stimulation over multiple sites, including bilateral median, ulnar, tibial, and peroneal nerves (Figure 2).

**Figure 3:**
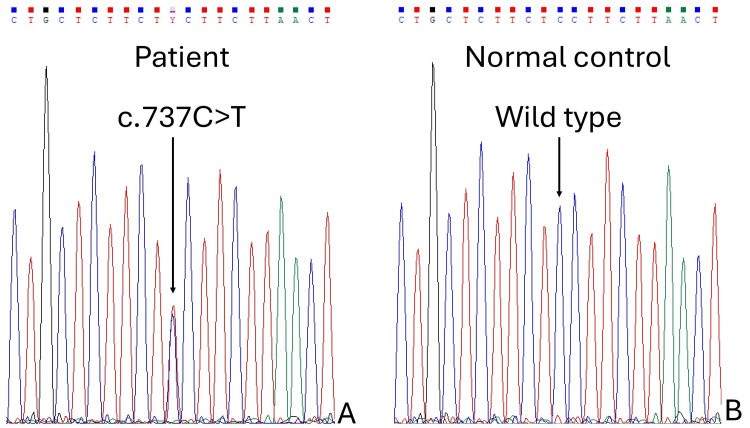
Sanger sequencing of CHRNA1 exon 6. (A) The patient was found to have heterozygous NM_000079.4(CHRNA1):c.737C>T p.(Ser246Phe) on Sanger sequencing. (B) Control patient showing wild-type sequence for comparison.

The patient was identified to have heterozygous NM_000079.4(*CHRNA1*):c.737C>T p.(Ser246Phe) (Figure 3) on cascade family screening for symptomatic family members using targeted genetic analysis by Sanger’s sequencing (the proband being her younger sister). The variant was previously reported as pathogenic for SCCMS by Ohno et al., who demonstrated prolonged activation responses to ACh in cells transfected with the variant [6]. The patient was subsequently started on fluoxetine 40mg daily for treatment of her SCCMS. Treatment progress could not be assessed as the patient defaulted on follow-up.

Family history

The patient’s younger sister, who presented earlier to our hospital at 27 years old for progressive fatigable generalized weakness since approximately 10 years of age, was also diagnosed with *CHRNA1*-related SCCMS. Examination of the younger sister revealed symmetrical weakness (MRC grade 4/5) over proximal upper limbs and lower limbs, bilateral partial ptosis with positive Cogan’s lid twitch, ophthalmoplegia, weakness of neck flexion and extension, weakness of facial muscles, as well as dysarthria with nasal speech. The younger sister had a low body weight but was not diagnosed with any eating disorders. Electrophysiological testing of the younger sister identified a repetitive CMAP upon single stimulation to the median nerve of the left wrist. Serology, including anti-AChR antibodies, was normal. Germline genetic analysis on the younger sister’s was performed using next-generation sequencing on Illumina NextSeq 2000 (Illumina, Inc., San Diego, CA) with library preparation kit Illumina DNA Prep with Enrichment and Illumina Exome Panel. She was found to have heterozygous NM_000079.4(*CHRNA1*):c.737C>T p.(Ser246Phe), a pathogenic variant for SCCMS.

The younger sister was started on fluoxetine 40mg daily, with remarkable improvement in muscle strength and endurance. Four weeks after fluoxetine treatment, her limb power was MRC grade 5/5, with only residual mild diplopia. She did not experience any adverse effects from fluoxetine.

The patient’s symptomatic father and asymptomatic mother did not undergo genetic testing, as they had passed away due to non-neurological illnesses. The patient’s symptomatic father was reported to be very thin, but had no formal diagnosis of any eating disorders.

## Discussion

CMS is a group of clinically heterogeneous inherited disorders characterized by problems of the neuromuscular junction, resulting in fatigable weakness in skeletal muscles. SCCMS are caused by pathogenic variants affecting the α, β, δ, or ε subunits of the AChR [7], which are encoded by the genes *CHRNA1*, *CHRNB1*, *CHRND*, and *CHRNE*, respectively.

SCCMS is characterized by abnormal kinetics of the AChR, resulting in prolonged activation of the AChR and subsequently slow decay of endplate potential [8]. Mutation of the AChR in SCCMS results in a decreased rate of dissociation of ACh from its receptor, causing repetitive opening during ACh occupancy [9]. Consequently, SCCMS can be differentiated from acquired autoimmune myasthenia gravis (MG) on EMG on neurophysiology testing by the presence of a repetitive muscle response to a single stimulus [2], as well as by increased duration and half-decay time of miniature endplate potential (MEPP) [1]. In vitro intracellular microelectrode studies identified prolonged MEPP and endplate potential (EPP) in SCCMS [1]. The addition of an AChE inhibitor further prolonged the opening duration [1], explaining the poor clinical response to AChE inhibitors in patients with SCCMS [3]. In SCCMS patients, loss of AChR in degenerating junctional folds is associated with muscle weakness severity [1].

The prolonged opening time of the ACh-induced ion channels results in greater Ca^2+^ flux into junctional folds and nearby muscle fiber regions, which overloads the Ca^2+^ uptake capability of the sarcoplasmic reticulum [1]. This results in inhibition of mitochondrial respiration [10], activation of intracellular proteases [11], and depolymerization of microtubules [12]. The role of focal Ca^2+^ overload in endplate degeneration is supported by observation of Ca^2+^ deposits at the neuromuscular junction in SCCMS [1].

The pathogenic role of the patient’s variant, heterozygous NM_000079.4(CHRNA1):c.737C>T p.(Ser246Phe), was identified by Ohno et al. [6]. Transfected HEK cells harboring the αS246F-AChR mutant demonstrated 12-fold prolonged activation episodes at low concentrations of ACh [6]. The mechanism of prolonged activation involved both decreased AChR channel closing rate and decreased dissociation rate of ACh from AChR [6].

The treatment of CMS depends on the subtype of CMS. In SCCMS, fluoxetine and quinidine consistently demonstrated benefit, while AChE inhibitors, such as pyridostigmine, showed no benefit or worsening of symptoms, and hence should be avoided in SCCMS patients [3,13]. While increasing synaptic acetylcholine with an AChE inhibitor improves weakness in autoimmune MG, the increased ACh may worsen the weakness by enhancing the depolarizing block in SCCMS [4]. On the other hand, medications that shorten the duration of channel opening while preserving the amplitude of miniature end-plate potentials have been frequently reported to be effective in improving muscle strength in SCCMS patients, occasionally with dramatic response [4]. Both fluoxetine and quinidine are long-acting open-channel blockers that shorten the duration of channel opening events in a concentration-dependent manner, and thus are recommended in the treatment of SCCMS [13].

To the best of our knowledge, we present the first case of CMS in which the patient’s low body weight resulted in a diagnosis of atypical anorexia nervosa. ICD-10 defined atypical anorexia nervosa (ICD-10 code F50.1) as “disorders that fulfill some of the features of anorexia nervosa but in which the overall clinical picture does not justify that diagnosis” [5]. The classification of atypical anorexia nervosa was removed in ICD-11.

AN is a multifactorial disorder with psycho-social contributions as well as biological factors, and the role of genetics in anorexia nervosa has been increasingly recognized. Case-control population-based analysis observed a threefold rise in the proportion of AN in those with positive family history [14]. The heritability estimate for AN obtained from twin studies ranges from 0.48 to 0.74 [15]. An adoption study identified that genetic factors, as opposed to environmental factors, accounted for the correlation of AN between siblings [16]. Genome-wide analysis of families with AN found modest evidence of linkage at marker D1S3721 on chromosome 1 and at marker D4S2367 on chromosome 4 [17]. Subsequent genome-wide association studies have identified many more association genes [18]. Further genetic research on genes associated with AN may improve the understanding of the pathological mechanism of AN, potentially facilitating the development of pharmacological treatments for anorexia nervosa [18]. Despite such advances, the role of genetic testing in treatment decisions is limited; hence, genetic testing remains uncommon in routine clinical practice in the treatment of anorexia nervosa.

We present a case where a patient’s SCCMS resulted in low body weight, which mimicked anorexia nervosa. The patient’s low body weight was postulated to be related to muscle wasting in addition to weakness of muscles of mastication, the latter of which contributed to the patient’s limited food intake and persistent low body weight despite the absence of a fear of gaining weight or any preoccupation with body shape. The diagnosis of the underlying condition was only identified serendipitously upon genetic testing during family cascade screening, with phenotypic confirmation on neurophysiology examination. While rare diseases are individually uncommon, rare diseases collectively constitute a significant disease burden. A high index of suspicion for neuromuscular disorders is required for the proper workup and diagnosis in patients with low body weight.

Neither the patient’s symptomatic father nor the younger sister had been diagnosed with eating disorders. SCCMS has been documented to demonstrate intra-familial phenotypic variability [19], a phenomenon that was also apparent in this family. The younger sister experienced ptosis as well as a symmetrical pattern of four-limb muscle weakness. In contrast, the patient had no ptosis, had asymmetrical weakness more prominent over the left distal lower limb, and experienced seasonal fluctuation of her symptoms.

While the patient and her symptomatic family members had low body weight, we have yet to delineate potential confounding factors from other genetic or environmental factors contributing to their low body weight. In addition, the exact mechanism by which SCCMS causes low body weight has yet to be confirmed. Further functional studies related to the muscles of mastication may unravel the underlying mechanisms. In addition, further population studies assessing the body weight in patients with CMS will be useful in assessing the prevalence of low body weight among CMS patients and in identifying genotype-phenotype associations. Long-term cohort studies on the body weight of these patients before and after pharmacological treatment of CMS may be conducted to explore the efficacy of pharmacological treatment on body weight gain in CMS patients with low body weight.

## Conclusions

We report a patient with extremely low body weight without engaging in dieting, purging, or excessive exercising. The patient demonstrated characteristic findings of repetitive CMAP on nerve stimulation and was identified to have the pathogenic variant of heterozygous NM_000079.4(*CHRNA1*):c.737C>T p.(Ser246Phe), which established the diagnosis of SCCMS. This case illustrates that low body weight could be a phenotype of SCCMS. Hence, genetic testing and electrophysiology studies for rare neuromuscular diseases should be considered for patients presenting with low body weight and suspected anorexia nervosa to facilitate the diagnosis of underlying physical conditions.
